# Sex Differences in Instrumental Activities of Daily Living and Transportation Modes by Driving Status Among Older Adults

**DOI:** 10.1155/oti/6621910

**Published:** 2026-05-09

**Authors:** Tsutomu Sasaki, Masao Ichikawa, Kyohei Yamada, Yukiko Tomari, Daiji Kobayashi

**Affiliations:** ^1^ Department of Occupational Therapy, Hokkaido Chitose College of Rehabilitation, Chitose City, Hokkaido, Japan; ^2^ Department of Global Public Health Policy, Institute of Medicine, University of Tsukuba, Tsukuba City, Ibaraki, Japan, tsukuba.ac.jp; ^3^ Shimamatsu Hospital, Eniwa City, Hokkaido, Japan; ^4^ Department of Information System Engineering, Faculty of Science and Technology, Chitose Institute of Science and Technology, Chitose City, Hokkaido, Japan

**Keywords:** ageing, community, driving, health, instrumental activities of daily living, sex

## Abstract

**Introduction:**

Planning for driving cessation is essential to support older adults’ well‐being and access to alternative transportation. As transportation needs vary by activity and sex, it is important to understand how these factors influence mobility after driving cessation. This study aimed to clarify sex differences in transportation modes for daily activities among older adults with different driving status.

**Materials and Methods:**

This study examined transportation modes used for instrumental activities of daily living (IADLs) and the level of engagement (i.e., frequency of participation) among community‐dwelling older adults, stratified by driving status (current, retired, never) and sex. Cross‐sectional data were obtained from a 2023 self‐administered survey of residents aged 75–80 years in Chitose City, Japan, including 916 current drivers, 238 retired drivers, and 281 never drivers.

**Results:**

Nearly all participants engaged in shopping and visiting medical facilities. Current drivers showed higher engagement in IADLs, primarily by driving themselves. Retired and never drivers engaged in fewer IADLs, particularly men, who were less likely to receive rides from others. In contrast, women were more often driven by others, especially among nondrivers. Men reported higher bicycle use across driving statuses. Multivariable analyses showed that driving status was strongly associated with essential IADLs, whereas discretionary IADLs were influenced by sex and social support.

**Discussion:**

These findings suggest that mobility support for older adults should consider both sex and driving status. Occupational therapists and policymakers should tailor interventions—such as safe bicycle use training and transportation support—to promote equitable community mobility. Supporting meaningful engagement in daily activities is vital for maintaining health, independence, and social participation after driving cessation.

## 1. Introduction

As the older adult population continues to increase, many countries face the social challenges of maintaining and improving the health and well‐being of older adults. One of the challenges is supporting their mobility. In many areas, the primary mode of transportation supporting their mobility is driving because alternative transportation is unavailable or inconvenient to use. Even if alternative transportation is available, the ability to drive a car symbolizes their independence and is linked to their health and well‐being [1–3]. Cohort studies targeting older adults have revealed that driving cessation can lead to an increased risk of social isolation [4, 5], depression [6, 7], cognitive and functional decline [8–10], institutionalization [11], and mortality [12]. Social isolation and loneliness, in particular, are considered key mechanisms linking reduced mobility to adverse health outcomes among older adults [4, 5]. The impact of driving cessation on health is also influenced by pre‐existing health conditions [13, 14] and residential location [15]. Importantly, the relationship between driving cessation and health is likely bidirectional: declining health may lead individuals to stop driving, while driving cessation itself may further contribute to reduced activity and worsening health outcomes. Additionally, there are sex differences in the impact of driving cessation, with men experiencing higher risks of social isolation [16], depression [7], and mortality [12] than women.

Adverse outcomes of driving cessation could be attenuated with social activities and mobility support. For example, participation in volunteering and maintaining connections with social support [17], as well as using public transportation to go out [9] may help reduce the risk of becoming dependent on care and maintain older adults’ well‐being. Receiving psychoeducational programs before or after deciding to cease driving could further alleviate the psychological conflict associated with driving cessation [18], enhance older adults’ ability to find alternative transportation modes [19], and increase their awareness of using public transportation [20], in which occupational therapists play crucial roles alongside other stakeholders such as physicians, social workers, and family members.

Advance planning and preparation for driving cessation is important for older drivers to maintain their well‐being, which is facilitated by using alternative transportation and remaining socially engaged. Supporting this process falls in the responsibilities of occupational therapists. Previous studies have reported that the availability and preferences for transportation modes [21–24], as well as the level of engagement (i.e., frequency of participation) with instrumental activities of daily living (IADL) [25–28] differed by sex. However, these studies did not consider driving status, which could largely influence IADLs such as shopping and hobbies. Moreover, few studies have examined how driving status interacts with sex to influence the frequency and patterns of engagement in IADLs while accounting for relevant covariates. To assist older drivers with their transition to non‐driving while remaining socially engaged, we need to understand the level of social engagement by driving status and what modes of transportation other than motor vehicles are used for it. Furthermore, understanding these relationships using detailed measures of engagement frequency may help clarify more nuanced patterns of activity participation. Accordingly, using baseline data from a prospective cohort study of community‐dwelling older adults, to clarify sex differences in the use of transportation modes for IADLs among older adults with different driving status, we analyzed the proportion of transportation modes used for several IADLs, as well as differences in perceptions of physical health, mental health, and life satisfaction at the baseline survey, among current, retired, and never drivers by sex. In addition, we explored factors associated with the frequency of engagement in IADLs using multivariable ordinal analyses to better capture variations in activity participation.

## 2. Materials and Methods

### 2.1. Study Design and Setting

This is a cross‐sectional study using baseline data from the Driving and Wellness cohort study, a three‐year prospective study investigating the relationship between driving status, health, and well‐being in a cohort of older adults residing in Chitose City, which is composed of five wards and located in the southwestern region of Hokkaido, Japan. This city has a population of approximately 98,000 in a land area of 594 km^2^, which is equivalent to the total land area of 23 special wards of Tokyo. This study, which did not involve clinical trials, was approved by the Research Ethics Committee of Hokkaido Chitose College of Rehabilitation (Approval Number R02303). The intent to participate in this study was confirmed by the return of the questionnaire and the written consent form.

### 2.2. Participants and Data Collection

The prospective participants were 5446 residents aged 75–80 years (2431 men, 3015 women) registered in the Basic Resident Register of Chitose City as of September 15, 2023. Individuals who were hospitalized or institutionalized and those who had passed away before the study began were excluded. On September 28, 2023, we mailed out self‐administered questionnaires to the remaining 5305 residents (2378 men, 2927 women) for data collection. By November 30, 2023, 1466 residents (775 men, 660 women, 31 sex unspecified) returned the questionnaires and 1435 (775 men and 660 women) were subjected to the analysis. The response rate for the five wards in the city ranged from 24% to 31%.

### 2.3. Measures

In the baseline questionnaire, we asked about the participant’s characteristics, including age, sex, number of household members, employment status, subjective physical and mental health (rated on 4‐point Likert scale), life satisfaction (rated on 4‐point Likert scale), health conditions under treatment, number of prescribed drugs, certification for long‐term care insurance benefits, driving status (current drivers, retired drivers [those who stopped driving], and never drivers [those who have never obtained a driver’s license]), transportation modes used for IADLs, and the frequency of their use.

The IADLs of interest included the following: (1) shopping for daily necessities, (2) visiting medical facilities, (3) leisure activities, (4) eating out, (5) visiting someone’s house, (6) engaging in hobbies, (7) overnight travel, (8) pick‐up/drop‐off, and (9) volunteering. We asked the participants to provide the frequency (not at all, rarely, 1–2 times a month, 1–2 times a week, 3–5 times a week, 6–7 times a week) of using six transportation modes (driving myself, driven by others, walking, public transportation [bus, subway, train], bicycle, and taxi) for each IADL.

### 2.4. Analysis

First, we described the participants’ baseline characteristics by sex and current, retired, and never drivers (to be followed in the next 3 years to investigate social and health outcomes by driving status). Subsequently, group comparisons were conducted by sex and driving status to examine differences in the distribution of subjective physical health, subjective mental health, life satisfaction, and transportation mode used for each IADL. Since the frequency of going out for IADLs depends on each individual IADL needs, in this baseline survey, we calculated the proportion of those using each transportation mode for each IADL, regardless of the frequency, to understand what mode of transportation can potentially be used for IADLs, depending on the current driving status.

In addition, to further explore factors associated with the frequency of engagement in IADLs, we conducted ordinal logistic regression analyses. The frequency of engagement in each IADL (not at all, rarely, 1–2 times a month, 1–2 times a week, 3–5 times a week, and 6–7 times a week) was treated as an ordinal outcome variable. Six separate models were constructed for the following IADLs: shopping for daily necessities, visiting medical facilities, leisure activities, eating out, visiting someone’s houses, and hobbies. Independent variables included driving status (current, retired, never), age, sex, household size, availability of family or acquaintances to drive (yes/no), need for long‐term care (yes/no), and the walkability index (WI) [29]. The WI is a measure of neighborhood walkability based on postal code areas in Japan, reflecting the accessibility of daily living facilities within walking distance; higher values indicate greater ease of walking for daily living. Furthermore, to consider differences in the nature of activities, IADLs were categorized into essential activities (shopping and visiting medical facilities) and discretionary activities (eating out, visiting others, leisure activities, and hobbies), and results were interpreted based on this classification. A cumulative logit link function was used for the ordinal logistic regression models, and the proportional odds assumption was assessed for each model and was not violated.

For statistical analysis, to account for multiple comparisons, the Benjamini–Hochberg (BH) procedure was applied to control the false discovery rate (FDR), and adjusted *p* values were calculated. For the ordinal logistic regression analyses, Holm correction was additionally applied to adjust for multiple testing across models. For each model, regression coefficients (*β*), standard errors (SE), *p* values, odds ratios (OR), and 95% confidence intervals (CI) were estimated. A significance threshold of 5% (adjusted *p* < 0.05) was used. For descriptive group comparisons, effect sizes for pairwise comparisons were estimated using Cliff’s delta and Cramer’s V; Cliff’s delta was used for ordinal variables, and Cramer’s V was used for differences in proportions. The magnitudes of effect sizes were interpreted based on established thresholds: for Cliff’s delta, |*δ*| < 0.147 was considered negligible, 0.147–0.33 small, 0.33–0.474 medium, and ≥ 0.474 large [30]; for Cramer’s V, values < 0.10 were considered negligible, 0.10–0.30 small, 0.30–0.50 medium, and ≥ 0.50 large [31]. All statistical analyses were performed using R version 4.4.3.

## 3. Results

Of the 1435 participants (mean age = 77 years [standard deviation = 1.8] ), 54% were men, 17% were living alone, 19% had a paid job, 76% had someone to ask for a ride, and 8% were certified long‐term care recipients. Table 1 shows the characteristics of the 916 current drivers (665 men, 251 women), 238 retired drivers (93 men, 145 women), and 281 never drivers (17 men, 264 women). The mean age was similar across these groups by driving status and sex. Women tended to live alone more often compared with men among current and retired drivers, but the opposite was true among never drivers. Current drivers tended to have a paid job for both sexes compared with retired and never drivers. A large majority of the participants had someone to ask for a ride, although men tended not to have one compared with women regardless of driving status: over 80% of women had one whereas 65% (61/93) of male retired drivers, 71% (471/665) of current drivers, and 77% (13/17) of never drivers did. Retired and never drivers were more likely to be certified for long‐term care compared with current drivers regardless of sex, and men were more likely than women to be certified for long‐term care regardless of driving status.

**Table 1:** Characteristics of current, retired, and never drivers by sex.

	Current drivers		Retired drivers		Never drivers	
	Men (*n* = 665)	Women (*n* = 251)	Men (*n* = 93)	Women (*n* = 145)	Men (*n* = 17)	Women (*n* = 264)
Mean age (±standard deviation)	77.0 ± 1.7	76.8 ± 1.6	77.6 ± 1.7	77.2 ± 1.8	77.5 ± 1.8	77.7 ± 1.8

Household size						
One person (living alone) (%)	11.4	20.3	21.5	27.6	35.3	18.6
Two people (%)	62.3	59.8	46.2	46.9	47.1	54.8
Three or more people (%)	26.2	19.9	32.3	25.5	17.6	26.6

Working for pay (%)	27.4	22.3	2.2	5.5	11.8	8.3
Availability of family or acquaintances to drive (%)	70.9	82.0	65.2	82.8	76.5	83.1
Certified long‐term care recipients (%)	3.2	2.0	22.6	13.8	41.2	14.6

Table 2 displays the level of subjective physical and mental health and life satisfaction by driving status and sex. Compared with retired and never drivers, current drivers had better perceptions of their physical and mental health and life satisfaction (adjusted *p* < 0.001 for all comparisons with retired and never drivers across each measure). The perceptions were generally similar between men and women among current drivers (adjusted *p* = 0.314–0.472, Cliff^’^s delta = 0.032–0.094), with negligible difference observed only for life satisfaction (adjusted *p* = 0.022, Cliff^’^s delta = 0.094). Among retired drivers, men tended to have worse perceptions compared with women, especially in life satisfaction (adjusted *p* = 0.001, Cliff^’^s delta = 0.248), with smaller differences in physical and mental health. A similar trend was observed among never drivers, particularly for physical health (adjusted *p* = 0.003, Cliff^’^s delta = 0.436) and life satisfaction (adjusted *p* = 0.134, Cliff^’^s delta = 0.217). Details of all comparisons are provided in Appendix A.

**Table 2:** Subjective physical and mental health and life satisfaction among current, retired, and never drivers by sex.

	Current drivers		Retired drivers		Never drivers	
	Men (*n* = 665)	Women (*n* = 251)	Men (*n* = 93)	Women (*n* = 145)	Men (*n* = 17)	Women (*n* = 264)
Subjective physical health (%)						
Healthy	27.8	32.8	16.1	15.2	0	19.2
Relatively healthy	56.3	51.2	32.3	52.4	29.4	47.3
Relatively unhealthy	13.4	14.4	35.5	20.7	47.1	23.5
Unhealthy	2.6	1.6	16.1	11.7	23.5	10.0

Subjective mental health (%)						
Healthy	49.0	52.2	28.0	31.7	17.6	28.8
Relatively healthy	44.2	41.4	37.6	46.2	64.7	49.6
Relatively unhealthy	6.1	6.4	28.0	18.6	17.6	17.3
Unhealthy	0.8	0	6.5	3.4	0	4.2

Life satisfaction (%)						
Satisfied	39.8	48.2	18.3	31.0	11.8	28.8
Relatively satisfied	51.6	458	44.1	51.7	58.8	52.1
Relatively dissatisfied	7.4	6.0	32.3	15.2	17.6	13.2
Dissatisfied	1.2	0	5.4	2.1	11.8	5.8

Table 3 shows the proportion of participants engaging in each IADL with each transportation mode among current, retired, and never drivers by sex. The vast majority of the participants, regardless of their driving status and sex, engaged in shopping for daily necessities, visited medical facilities, and engaged in leisure activities. Current drivers were more likely to engage in each IADL, except for visiting medical facilities, than retired and never drivers, regardless of sex (adjusted *p* < 0.05). Among current drivers, women showed higher engagement in shopping and visiting someone’s houses, while men were more engaged in other IADLs. In contrast, for both retired and never drivers, women tended to engage more than men across all IADLs, though none of the differences were statistically significant (adjusted *p* > 0.05).

**Table 3:** Number and proportion of participants engaging in instrumental activities of daily living (IADLs) and proportion of those using each transportation mode for each IADL among current, retired, and never drivers by sex.

	Current drivers		Retired drivers		Never drivers	
	Men (*n* = 665)	Women (*n* = 251)	Men (*n* = 93)	Women (*n* = 145)	Men (*n* = 17)	Women (*n* = 264)
Shopping for daily necessities	646 (97%)	245 (98%)	80 (86%)	138 (95%)	15 (88%)	253 (96%)
Driving myself	98%	99%				
Driven by others	42%	56%	68%	86%	80%	89%
Walking	68%	55%	88%	80%	73%	76%
Public transportation	14%	17%	53%	50%	40%	49%
Bicycle	32%	9%	46%	21%	47%	21%
Taxi	4%	4%	38%	33%	47%	36%

Visiting medical facilities	621 (93%)	229 (91%)	86 (92%)	135 (93%)	16 (94%)	238 (90%)
Driving myself	96%	95%				
Driven by others	36%	35%	72%	68%	81%	79%
Walking	30%	28%	65%	60%	50%	59%
Public transportation	25%	30%	59%	54%	63%	54%
Bicycle	13%	6%	33%	14%	25%	17%
Taxi	16%	20%	44%	45%	50%	53%

Leisure activities	576 (86%)	208 (83%)	71 (76%)	124 (86%)	13 (76%)	210 (80%)
Driving myself (for driving)	80%	64%				
Driven by others (for driving)	39%	55%	24%	66%	31%	69%
Walking (for strolling)	84%	80%	92%	85%	85%	87%
Public transportation (for a day trip)	24%	27%	21%	24%	15%	24%
Bicycle (for cycling)	17%	6%	24%	6%	23%	8%
Taxi (for a day trip)	8%	4%	6%	4%	8%	6%

Eating out	533 (80%)	198 (79%)	53 (57%)	105 (72%)	10 (59%)	180 (68%)
Driving myself	93%	90%				
Driven by others	53%	66%	74%	87%	80%	92%
Walking	28%	21%	51%	50%	80%	44%
Public transportation	19%	14%	45%	42%	40%	38%
Bicycle	5%	1%	25%	8%	30%	9%
Taxi	16%	11%	21%	17%	30%	23%

Visiting someone’s houses	548 (82%)	222 (88%)	47 (51%)	96 (66%)	8 (47%)	186 (70%)
Driving myself	95%	86%				
Driven by others	49%	55%	72%	78%	63%	82%
Walking	44%	47%	60%	70%	63%	77%
Public transportation	16%	17%	43%	41%	50%	40%
Bicycle	20%	9%	34%	18%	50%	17%
Taxi	8%	4%	28%	24%	25%	22%

Going out for hobby activities	499 (75%)	179 (71%)	37 (40%)	79 (54%)	5 (29%)	131 (50%)
Driving myself	96%	98%				
Driven by others	42%	40%	62%	66%	80%	81%
Walking	52%	33%	70%	68%	40%	73%
Public transportation	21%	21%	49%	42%	80%	43%
Bicycle	18%	6%	38%	15%	40%	18%
Taxi	7%	6%	19%	19%	20%	23%

Overnight travel	405 (61%)	147 (59%)	31 (33%)	69 (48%)	5 (29%)	108 (41%)
Driving myself	95%	71%				
Driven by others	52%	64%	65%	81%	80%	92%
Walking	14%	9%	35%	33%	40%	34%
Public transportation	41%	42%	61%	42%	60%	45%
Bicycle	2%	1%	3%	1%	20%	2%
Taxi	16%	16%	13%	16%	40%	17%

Pick‐up/drop‐off	521 (78%)	195 (78%)	18 (19%)	29 (20%)	3 (18%)	56 (21%)
Driving myself	96%	94%				
Driven by others	50%	55%	50%	69%	100%	75%
Walking	17%	12%	33%	10%	67%	34%
Public transportation	8%	8%	11%	2%	33%	20%
Bicycle	2%	1%	6%	0%	0%	7%
Taxi	6%	4%	39%	28%	33%	30%

Volunteering	263 (40%)	84 (33%)	13 (14%)	23 (16%)	5 (29%)	53 (20%)
Driving myself	86%	93%				
Driven by others	38%	43%	69%	57%	60%	75%
Walking	64%	45%	92%	83%	100%	83%
Public transportation	19%	21%	46%	48%	40%	34%
Bicycle	22%	11%	38%	17%	40%	13%
Taxi	7%	8%	8%	26%	20%	15%

Regarding transportation mode for the IADLs, current drivers tended to drive a car for all IADLs, with nearly 100% of both men and women driving for shopping and approximately 90% for visiting medical facilities, eating out, pick‐up/drop‐off, visiting someone’s house, and engaging in hobbies. Male current drivers were more likely to use a car for leisure activities (adjusted *p* < 0.001, Cramer^’^s V = 0.162), visiting someone’s houses (adjusted *p* < 0.001, Cramer^’^s V = 0.145) and overnight travel (adjusted *p* < 0.001, Cramer^’^s V = 0.325) than their female counterparts, whereas the opposite trend was observed for volunteering, although the difference was not statistically significant (men: 86%, women: 93%; adjusted *p* = 0.274, Cramer^’^s V = 0.094). Retired and never drivers tended to be driven by others (i.e., passive transportation) but walking and public transportation (i.e., active transportation) were also common modes of transportation. Regardless of driving status, men were more likely to use bicycles than women for various IADLs. For example, among current drivers, 32% of men used bicycles for shopping compared with 9% of women (adjusted *p* < 0.001, Cramer^’^s V = 0.238). A similar difference was observed among retired drivers (men: 46%, women: 21%; adjusted *p* = 0.001, Cramer^’^s V = 0.265).Women were more likely to be driven by others than men, even if they still drove themselves. For example, 42% of male current drivers were driven by others for shopping compared with 56% of female current drivers (adjusted *p* = 0.004, Cramer^’^s V = 0.119). Pick‐up/drop‐off was mostly done with cars. Details of all comparisons are provided in Appendix B.

Table 4 presents the results of the ordinal logistic regression analyses examining factors associated with the frequency of engagement in IADLs. For essential IADLs (shopping and visiting medical facilities), driving status was significantly associated with higher frequency of engagement, with current drivers showing higher frequencies than retired and never drivers after adjustment for covariates. Age was inversely associated with engagement frequency. Sex was not significantly associated with these essential activities after adjustment. For discretionary IADLs (leisure, eating out, visiting others, and hobbies), different patterns were observed. Sex was significantly associated with engagement frequency in several activities, with distinct patterns between men and women depending on the IADL. In addition, the availability of family or acquaintances to provide rides was positively associated with higher frequency of engagement. Household size and need for long‐term care were also associated with engagement in several discretionary activities. Across multiple models, the availability of family or acquaintances to provide rides was consistently associated with higher frequency of engagement, particularly for discretionary IADLs. Overall, driving status was primarily associated with essential IADLs, whereas discretionary IADLs were associated with a broader range of factors, including sex and social support. Full regression results, including regression coefficients and standard errors, are provided in Appendix C.

**Table 4:** Ordinal logistic regression models (cumulative logit) were used. Odds ratios represent the likelihood of higher frequency of engagement in each IADL. All models were adjusted for driving status, age, sex, household size, availability of family or acquaintances to provide rides, need for long‐term care, and walkability index. *p* values were adjusted using the Holm method to account for multiple testing across models. Reference categories were as follows: current drivers for driving status, women for sex, absence of available drivers for ride support, and no need for long‐term care. OR, odds ratio; CI, confidence interval; IADL, instrumental activities of daily living.

Variables	Shopping OR (95% CI)	*p*(Holm)	Hospital visit OR (95% CI)	*p*(Holm)	Leisure OR (95% CI)	*p*(Holm)	Eating out OR (95% CI)	*p*(Holm)	Visiting others OR (95% CI)	*p*(Holm)	Hobby OR (95% CI)	*p*(Holm)
Driving status: retired vs. current (ref: current)	0.47 (0.35, 0.63)	< 0.001	1.07 (0.80, 1.44)	0.657	1.08 (0.82, 1.42)	0.574	0.58 (0.43, 0.77)	< 0.001	0.30 (0.23, 0.41)	< 0.001	0.41 (0.31, 0.55)	< 0.001
Driving status: never vs. current (ref: current)	0.54 (0.40, 0.73)	< 0.001	0.90 (0.66, 1.24)	0.524	1.07 (0.80, 1.42)	0.668	0.56 (0.41, 0.75)	< 0.001	0.46 (0.34, 0.63)	< 0.001	0.37 (0.27, 0.50)	< 0.001
Age (per 1 year)	0.90 (0.85, 0.95)	< 0.001	1.03 (0.97, 1.10)	0.286	0.93 (0.88, 0.98)	0.005	0.95 (0.90, 1.01)	0.08	0.94 (0.89, 0.99)	0.029	1.03 (0.97, 1.09)	0.347
Sex: men vs. women (ref: women)	0.83 (0.66, 1.05)	0.116	0.92 (0.72, 1.17)	0.475	1.40 (1.12, 1.75)	0.003	0.85 (0.67, 1.07)	0.156	0.61 (0.48, 0.77)	< 0.001	0.82 (0.65, 1.03)	0.085
Household size (per 1 person)	1.00 (0.89, 1.11)	0.844	1.02 (0.91, 1.14)	0.778	0.95 (0.86, 1.05)	0.327	0.88 (0.79, 0.98)	0.022	0.92 (0.83, 1.03)	0.135	0.90 (0.81, 1.00)	0.061
Availability of family or acquaintances to drive: yes vs. no (ref: no)	0.83 (0.65, 1.05)	0.111	0.94 (0.74, 1.21)	0.643	1.37 (1.08, 1.72)	0.009	1.74 (1.37, 2.22)	< 0.001	2.02 (1.59, 2.57)	< 0.001	1.30 (1.03, 1.65)	0.027
Long‐term care: yes vs. no (ref: no)	0.33 (0.22, 0.49)	< 0.001	2.42 (1.61, 3.64)	< 0.001	0.67 (0.46, 0.97)	0.033	0.84 (0.58, 1.22)	0.363	0.56 (0.38, 0.84)	0.004	0.54 (0.36, 0.80)	0.002
Walkability index (per 1 unit)	1.59 (1.25, 2.02)	< 0.001	1.25 (0.98, 1.61)	0.077	1.36 (1.08, 1.72)	0.009	1.47 (1.15, 1.87)	0.002	1.22 (0.95, 1.56)	0.119	0.92 (0.74, 1.16)	0.493

## 4. Discussion

The present study involving community‐dwelling people aged 75–80 years revealed that almost all participants went shopping and visited medical facilities in their daily lives, but current drivers engaged in other IADLs more often than retired and never drivers. Among retired and never drivers, women engaged in these IADLs more often than men. Retired and never drivers used both active and passive transportation modes, depending on the IADL. Generally, women tended to be driven by others compared with men. Regardless of driving status, bicycle use was more common among men than women. Additionally, current drivers had better subjective health and life satisfaction than retired and never drivers.

The level of engagement in IADLs differed by sex and driving status. The level was lower for men and retired and never drivers than women and current drivers, possibly because a relatively large proportion of male retired and never drivers were certified for long‐term care. Moreover, social networks among men are generally not as wide as among women [32], thus, men might have limited opportunities to engage in activities. Reduced engagement in IADLs may also contribute to social isolation and loneliness [4, 5], which are known to be associated with adverse health outcomes among older adults. Nevertheless, the level of engagement was comparable between men and women among current drivers, which implies that driving helps people engage in the wide range of activities, which might be especially true for men.

The transportation modes used by men and women among retired and never drivers differed. Men used bicycles more often than women, potentially because men are less likely than women to perceive riding a bicycle as insecure [33, 34]. Another potential reason is their preference for not being dependent on others [35]. Moreover, men were less likely to have someone to ask for a ride than women, and thus might need to use active transportation. On the other hand, women tended to be driven by others, possibly for the opposite reason. Additionally, generational and cultural factors may have influenced these observed sex differences [28]. For example, older women in this age cohort may have had fewer opportunities to obtain a driver’s license or may have been less encouraged to drive compared with men. Similarly, cultural expectations regarding transportation modes, such as cycling, may differ by sex. Although these factors were not directly assessed in the present study, they should be considered as potential alternative explanations and warrant further investigation.

These findings would offer important implications for occupational therapy practice, particularly in supporting community mobility and participation in older adults suggested by Marfeo et al. [36]. Given that retired and never drivers, especially men, showed lower IADL engagement, occupational therapists should assess physical and cognitive functions, transportation access, and social resources. Men’s greater reliance on bicycles suggests the importance of evaluating balance, strength, and safety. Occupational therapists could introduce electric‐assist or three‐wheeled bicycles and provide relevant training. For women, who were often driven by others, support may focus on building transportation networks and promoting safe use of public transit. In Japan, occupational therapists are often involved through healthcare services, community‐based care systems, and preventive care programs, where they work collaboratively with physicians, social workers, and care managers. In these contexts, OTs contribute by assessing functional abilities, identifying potential risks in mobility, and facilitating discussions with older adults and their families regarding feasible and safe transportation options. Importantly, decisions regarding transportation and driving cessation are highly personal and may involve emotional, social, and practical considerations. Therefore, individuals may not always be receptive to direct recommendations from healthcare professionals. In this context, the role of OTs is not to unilaterally direct decisions but to support shared decision‐making by providing individualized information, increasing awareness of alternative options, and aligning transportation choices with the person’s functional capacity and life context. In addition to occupational therapists, interdisciplinary collaboration with physicians, social workers, and family members is essential to support the transition from driving to non‐driving and to maintain community participation. Tailored interventions by sex and driving status are essential.

Subjective health and life satisfaction were lower among retired and never drivers than among current drivers. Moreover, men had a lower perception than women among retired and never drivers, which was not the case among current drivers. However, given the cross‐sectional nature of the baseline data, the direction of this association remains unclear. It is possible that poorer health led individuals to cease driving, while driving cessation itself may further contribute to reduced activity levels and poorer health outcomes. Based on the cross‐sectional baseline data, it is uncertain whether this is the consequence of driving cessation or associated with their functional disabilities or limited IADLs, which differed by sex. These findings should therefore be interpreted as associations rather than causal relationships. In our prospective cohort study, we will examine the trajectory of subjective health and life satisfaction in relation to driving status and IADLs and identify potential buffers for the adverse social and health outcomes of driving cessation.

## 5. Limitations

This study has several limitations. First, our findings could be distorted if the level of engagement in IADLs by driving status among nonrespondents largely differs from that of respondents. However, there were no substantial differences in the response rate across the five wards of the city that might possibly influence the level of engagement in IADLs by driving status because of different walkability and accessibility to public transportation in the wards. Second, the number of retired and never drivers, especially among men, was small, as was the number of those engaging in IADLs. In particular, the small sample size of male never drivers may have limited the statistical power and stability of the estimates, and may have influenced the observed sex differences within this subgroup. Therefore, the findings related to male never drivers should be interpreted with caution, and generalization to the broader population is limited. Third, we only described what transportation modes were used for each IADL among current, retired, and never drivers by sex to understand the availability and preferences of transportation modes, depending on their driving status. However, underlying factors for their choice of transportation (e.g., health status, family situation, and access to public transportation) remain to be investigated. Additionally, although we adjusted for several relevant covariates, residual confounding due to unmeasured factors cannot be ruled out.

## 6. Conclusion

Current drivers were more likely to engage in various IADLs than retired and never drivers, mostly by driving themselves. Regardless of driving status, men used bicycles more often than women. Among retired and never drivers, passive transportation was more common among women than men. Men were less likely to have someone to ask for a ride than women. Occupational therapists should consider different activity levels by driving status and different transportation use, preferences, or availability by sex when supporting the active life and mobility of older adults, while taking into account individual contexts and the noncausal nature of the present findings.

## Funding

This study was supported by JSPS KAKENHI, 23K10537.

## Conflicts of Interest

The authors declare no conflicts of interest.

## Supporting Information

Additional supporting information can be found online in the Supporting Information section.

## Supporting information


**Supporting Information 1** Appendix A: Group comparisons in subjective physical and mental health and life satisfaction by driving status and sex.

**Supporting Information 2** Appendix B: Group comparisons in transportation mode used for selected instrumental activities of daily living by driving status and sex.

**Supporting Information 3** Appendix C: Multivariable ordinal logistic regression analyses of IADL engagement frequency.

## Data Availability

The data that support the findings of this study are available from the corresponding author on reasonable request.
