# Supplementary material for: Sex Differences in Instrumental Activities of Daily Living and Transportation Modes by Driving Status Among Older Adults
Source: Occup Ther Int. 2026 May 9;2026:6621910. doi: 10.1155/oti/6621910 (PMC13156801; doi:10.1155/oti/6621910)
Supplement: Supplementary file 1 — Supporting Information 1 Appendix A: Group comparisons in subjective physical and mental health and life satisfaction by driving status and sex. [file OTI-2026-6621910-s001.docx]

Appendix A. Group comparisons in subjective physical and mental health and life satisfaction by driving status and sex.

|  | Subjective physical health | |  | Subjective mental health | |  | Life satisfaction | |
| --- | --- | --- | --- | --- | --- | --- | --- | --- |
|  | Effect size | adjusted p value |  | Effect size | adjusted p value |  | Effect size | adjusted p value |
| Current vs. Retired drivers | 0.296 | < 0.001 |  | 0.277 | < 0.001 |  | 0.248 | < 0.001 |
| Current vs. Never drivers | 0.242 | < 0.001 |  | 0.286 | < 0.001 |  | 0.203 | < 0.001 |
| Retired vs. Never drivers | 0.055 | 0.322 |  | 0.023 | 0.630 |  | 0.044 | 0.390 |
|  |  |  |  |  |  |  |  |  |
| Male vs. Female current drivers | 0.044 | 0.314 |  | 0.032 | 0.472 |  | 0.094 | 0.022 |
| Male vs. Female retired drivers | 0.148 | 0.059 |  | 0.116 | 0.139 |  | 0.248 | 0.001 |
| Male vs. Female never drivers | 0.436 | 0.003 |  | 0.053 | 0.725 |  | 0.217 | 0.134 |

Effect size (Cliff’s delta) of <0.147 indicates negligible, <0.330 small, <0.474 medium, and ≧0.474 large differences.
