# Supplementary material for: Sex Differences in Instrumental Activities of Daily Living and Transportation Modes by Driving Status Among Older Adults
Source: Occup Ther Int. 2026 May 9;2026:6621910. doi: 10.1155/oti/6621910 (PMC13156801; doi:10.1155/oti/6621910)
Supplement: Supplementary file 2 — Supporting Information 2 Appendix B: Group comparisons in transportation mode used for selected instrumental activities of daily living by driving status and sex. [file OTI-2026-6621910-s002.docx]

Appendix B. Group comparisons in transportation mode used for selected instrumental activities of daily living by driving status and sex.

|  |  | All modes combined | |  | Driving myself | |  | Driven by others | |  | Walking | |  | Public transportation | |  | Bicycle | |  | Taxi | |
| --- | --- | --- | --- | --- | --- | --- | --- | --- | --- | --- | --- | --- | --- | --- | --- | --- | --- | --- | --- | --- | --- |
|  |  | Effect size | adjusted p value |  | Effect size | adjusted p value |  | Effect size | adjusted p value |  | Effect size | adjusted p value |  | Effect size | adjusted p value |  | Effect size | adjusted p value |  | Effect size | adjusted p value |
| Shopping for daily necessities | |  |  |  |  |  |  |  |  |  |  |  |  |  |  |  |  |  |  |  |  |
|  | Current vs. Retired drivers | 0.119 | < 0.001 |  |  |  |  | 0.149 | < 0.001 |  | 0.078 | 0.001 |  | 0.232 | < 0.001 |  | 0.025 | 0.360 |  | 0.286 | < 0.001 |
|  | Current vs. Never drivers | 0.046 | 0.169 |  |  |  |  | 0.200 | < 0.001 |  | 0.052 | 0.025 |  | 0.227 | < 0.001 |  | 0.023 | 0.401 |  | 0.300 | < 0.001 |
|  | Retired vs. Never drivers | 0.077 | 0.133 |  |  |  |  | 0.048 | 0.228 |  | 0.035 | 0.401 |  | 0.014 | 0.738 |  | 0.058 | 0.122 |  | 0.012 | 0.778 |
|  |  |  |  |  |  |  |  |  |  |  |  |  |  |  |  |  |  |  |  |  |  |
|  | Male vs. Female current drivers | 0.013 | 0.853 |  | 0.017 | 0.835 |  | 0.119 | 0.004 |  | 0.121 | 0.004 |  | 0.037 | 0.559 |  | 0.238 | < 0.001 |  | 0.003 | 0.964 |
|  | Male vs. Female retired drivers | 0.161 | 0.134 |  |  |  |  | 0.213 | 0.014 |  | 0.099 | 0.414 |  | 0.024 | 0.914 |  | 0.265 | 0.001 |  | 0.050 | 0.758 |
|  | Male vs. Female never drivers | 0.086 | 0.337 |  |  |  |  | 0.064 | 0.612 |  | 0.014 | 0.947 |  | 0.041 | 0.770 |  | 0.142 | 0.101 |  | 0.053 | 0.720 |
|  |  |  |  |  |  |  |  |  |  |  |  |  |  |  |  |  |  |  |  |  |  |
| Visiting medical facilities | |  |  |  |  |  |  |  |  |  |  |  |  |  |  |  |  |  |  |  |  |
|  | Current vs. Retired drivers | 0.001 | 0.974 |  |  |  |  | 0.168 | < 0.001 |  | 0.172 | < 0.001 |  | 0.165 | < 0.001 |  | 0.086 | < 0.001 |  | 0.177 | < 0.001 |
|  | Current vs. Never drivers | 0.038 | 0.254 |  |  |  |  | 0.216 | < 0.001 |  | 0.159 | < 0.001 |  | 0.163 | < 0.001 |  | 0.060 | 0.010 |  | 0.224 | < 0.001 |
|  | Retired vs. Never drivers | 0.044 | 0.405 |  |  |  |  | 0.047 | 0.252 |  | 0.020 | 0.651 |  | 0.008 | 0.863 |  | 0.030 | 0.486 |  | 0.044 | 0.289 |
|  |  |  |  |  |  |  |  |  |  |  |  |  |  |  |  |  |  |  |  |  |  |
|  | Male vs. Female current drivers | 0.037 | 0.505 |  | 0.034 | 0.643 |  | 0.003 | 0.964 |  | 0.012 | 0.914 |  | 0.049 | 0.414 |  | 0.101 | 0.024 |  | 0.042 | 0.509 |
|  | Male vs. Female retired drivers | 0.012 | 0.887 |  |  |  |  | 0.042 | 0.801 |  | 0.051 | 0.758 |  | 0.051 | 0.758 |  | 0.220 | 0.010 |  | 0.010 | 0.954 |
|  | Male vs. Female never drivers | 0.032 | 0.839 |  |  |  |  | 0.016 | 0.944 |  | 0.043 | 0.764 |  | 0.041 | 0.790 |  | 0.049 | 0.758 |  | 0.012 | 0.951 |
|  |  |  |  |  |  |  |  |  |  |  |  |  |  |  |  |  |  |  |  |  |  |
| Leisure activities | |  |  |  |  |  |  |  |  |  |  |  |  |  |  |  |  |  |  |  |  |
|  | Current vs. Retired drivers | 0.041 | 0.229 |  |  |  |  | 0.037 | 0.181 |  | 0.017 | 0.599 |  | 0.013 | 0.655 |  | 0.011 | 0.717 |  | 0.027 | 0.360 |
|  | Current vs. Never drivers | 0.072 | 0.024 |  |  |  |  | 0.115 | < 0.001 |  | 0.017 | 0.599 |  | 0.012 | 0.672 |  | 0.044 | 0.092 |  | 0.014 | 0.651 |
|  | Retired vs. Never drivers | 0.032 | 0.565 |  |  |  |  | 0.088 | 0.023 |  | 0.001 | 0.984 |  | 0.002 | 0.969 |  | 0.043 | 0.345 |  | 0.019 | 0.672 |
|  |  |  |  |  |  |  |  |  |  |  |  |  |  |  |  |  |  |  |  |  |  |
|  | Male vs. Female current drivers | 0.048 | 0.337 |  | 0.162 | < 0.001 |  | 0.142 | 0.001 |  | 0.052 | 0.414 |  | 0.030 | 0.748 |  | 0.145 | 0.001 |  | 0.063 | 0.269 |
|  | Male vs. Female retired drivers | 0.116 | 0.245 |  |  |  |  | 0.406 | < 0.001 |  | 0.099 | 0.446 |  | 0.035 | 0.835 |  | 0.252 | 0.005 |  | 0.037 | 0.835 |
|  | Male vs. Female never drivers | 0.018 | 0.857 |  |  |  |  | 0.190 | 0.031 |  | 0.018 | 0.943 |  | 0.047 | 0.764 |  | 0.123 | 0.259 |  | 0.020 | 0.942 |
|  |  |  |  |  |  |  |  |  |  |  |  |  |  |  |  |  |  |  |  |  |  |
| Eating out | |  |  |  |  |  |  |  |  |  |  |  |  |  |  |  |  |  |  |  |  |
|  | Current vs. Retired drivers | 0.129 | < 0.001 |  |  |  |  | 0.106 | < 0.001 |  | 0.128 | < 0.001 |  | 0.158 | < 0.001 |  | 0.103 | < 0.001 |  | 0.026 | 0.401 |
|  | Current vs. Never drivers | 0.123 | < 0.001 |  |  |  |  | 0.152 | < 0.001 |  | 0.112 | < 0.001 |  | 0.135 | < 0.001 |  | 0.072 | 0.005 |  | 0.065 | 0.011 |
|  | Retired vs. Never drivers | 0.013 | 0.828 |  |  |  |  | 0.047 | 0.345 |  | 0.025 | 0.637 |  | 0.032 | 0.531 |  | 0.035 | 0.486 |  | 0.043 | 0.392 |
|  |  |  |  |  |  |  |  |  |  |  |  |  |  |  |  |  |  |  |  |  |  |
|  | Male vs. Female current drivers | 0.014 | 0.853 |  | 0.056 | 0.392 |  | 0.117 | 0.014 |  | 0.073 | 0.199 |  | 0.058 | 0.356 |  | 0.098 | 0.052 |  | 0.072 | 0.212 |
|  | Male vs. Female retired drivers | 0.159 | 0.134 |  |  |  |  | 0.162 | 0.183 |  | 0.013 | 0.954 |  | 0.032 | 0.897 |  | 0.235 | 0.024 |  | 0.044 | 0.826 |
|  | Male vs. Female never drivers | 0.048 | 0.674 |  |  |  |  | 0.098 | 0.450 |  | 0.162 | 0.123 |  | 0.010 | 0.954 |  | 0.157 | 0.136 |  | 0.035 | 0.835 |
|  |  |  |  |  |  |  |  |  |  |  |  |  |  |  |  |  |  |  |  |  |  |
| Visiting someone's houses | |  |  |  |  |  |  |  |  |  |  |  |  |  |  |  |  |  |  |  |  |
|  | Current vs. Retired drivers | 0.239 | < 0.001 |  |  |  |  | 0.109 | < 0.001 |  | 0.092 | < 0.001 |  | 0.145 | < 0.001 |  | 0.044 | 0.105 |  | 0.153 | < 0.001 |
|  | Current vs. Never drivers | 0.161 | < 0.001 |  |  |  |  | 0.140 | < 0.001 |  | 0.146 | < 0.001 |  | 0.154 | < 0.001 |  | 0.013 | 0.655 |  | 0.135 | < 0.001 |
|  | Retired vs. Never drivers | 0.094 | 0.060 |  |  |  |  | 0.024 | 0.651 |  | 0.053 | 0.276 |  | 0.003 | 0.957 |  | 0.041 | 0.401 |  | 0.027 | 0.611 |
|  |  |  |  |  |  |  |  |  |  |  |  |  |  |  |  |  |  |  |  |  |  |
|  | Male vs. Female current drivers | 0.074 | 0.134 |  | 0.145 | 0.001 |  | 0.071 | 0.201 |  | 0.026 | 0.758 |  | 0.012 | 0.915 |  | 0.155 | < 0.001 |  | 0.087 | 0.081 |
|  | Male vs. Female retired drivers | 0.156 | 0.134 |  |  |  |  | 0.064 | 0.758 |  | 0.102 | 0.509 |  | 0.018 | 0.947 |  | 0.182 | 0.136 |  | 0.040 | 0.835 |
|  | Male vs. Female never drivers | 0.121 | 0.166 |  |  |  |  | 0.097 | 0.450 |  | 0.070 | 0.646 |  | 0.039 | 0.826 |  | 0.172 | 0.087 |  | 0.017 | 0.947 |
|  |  |  |  |  |  |  |  |  |  |  |  |  |  |  |  |  |  |  |  |  |  |
| Going out for hobby activities | |  |  |  |  |  |  |  |  |  |  |  |  |  |  |  |  |  |  |  |  |
|  | Current vs. Retired drivers | 0.221 | < 0.001 |  |  |  |  | 0.097 | < 0.001 |  | 0.090 | 0.001 |  | 0.123 | < 0.001 |  | 0.050 | 0.087 |  | 0.102 | < 0.001 |
|  | Current vs. Never drivers | 0.233 | < 0.001 |  |  |  |  | 0.171 | < 0.001 |  | 0.108 | < 0.001 |  | 0.130 | < 0.001 |  | 0.030 | 0.345 |  | 0.136 | < 0.001 |
|  | Retired vs. Never drivers | 0.003 | 0.974 |  |  |  |  | 0.084 | 0.107 |  | 0.016 | 0.778 |  | 0.001 | 0.984 |  | 0.027 | 0.651 |  | 0.031 | 0.612 |
|  |  |  |  |  |  |  |  |  |  |  |  |  |  |  |  |  |  |  |  |  |  |
|  | Male vs. Female current drivers | 0.038 | 0.505 |  | 0.050 | 0.465 |  | 0.018 | 0.835 |  | 0.167 | < 0.001 |  | 0.006 | 0.954 |  | 0.145 | 0.002 |  | 0.022 | 0.823 |
|  | Male vs. Female retired drivers | 0.143 | 0.134 |  |  |  |  | 0.036 | 0.901 |  | 0.019 | 0.947 |  | 0.065 | 0.764 |  | 0.253 | 0.042 |  | 0.001 | 0.993 |
|  | Male vs. Female never drivers | 0.096 | 0.286 |  |  |  |  | 0.004 | 0.978 |  | 0.140 | 0.339 |  | 0.141 | 0.334 |  | 0.104 | 0.509 |  | 0.013 | 0.954 |
|  |  |  |  |  |  |  |  |  |  |  |  |  |  |  |  |  |  |  |  |  |  |
| Overnight travel | |  |  |  |  |  |  |  |  |  |  |  |  |  |  |  |  |  |  |  |  |
|  | Current vs. Retired drivers | 0.149 | < 0.001 |  |  |  |  | 0.083 | 0.006 |  | 0.145 | < 0.001 |  | 0.028 | 0.437 |  | 0.011 | 0.750 |  | 0.005 | 0.901 |
|  | Current vs. Never drivers | 0.171 | < 0.001 |  |  |  |  | 0.147 | < 0.001 |  | 0.153 | < 0.001 |  | 0.020 | 0.602 |  | 0.025 | 0.486 |  | 0.013 | 0.709 |
|  | Retired vs. Never drivers | 0.018 | 0.795 |  |  |  |  | 0.077 | 0.196 |  | 0.003 | 0.963 |  | 0.012 | 0.863 |  | 0.015 | 0.813 |  | 0.025 | 0.700 |
|  |  |  |  |  |  |  |  |  |  |  |  |  |  |  |  |  |  |  |  |  |  |
|  | Male vs. Female current drivers | 0.021 | 0.779 |  | 0.325 | < 0.001 |  | 0.107 | 0.069 |  | 0.064 | 0.395 |  | 0.006 | 0.954 |  | 0.039 | 0.696 |  | 0.002 | 0.978 |
|  | Male vs. Female retired drivers | 0.141 | 0.134 |  |  |  |  | 0.180 | 0.268 |  | 0.021 | 0.947 |  | 0.178 | 0.268 |  | 0.059 | 0.823 |  | 0.039 | 0.900 |
|  | Male vs. Female never drivers | 0.056 | 0.602 |  |  |  |  | 0.084 | 0.700 |  | 0.025 | 0.943 |  | 0.060 | 0.790 |  | 0.232 | 0.078 |  | 0.126 | 0.450 |
|  |  |  |  |  |  |  |  |  |  |  |  |  |  |  |  |  |  |  |  |  |  |
| Pick-up/drop-off | |  |  |  |  |  |  |  |  |  |  |  |  |  |  |  |  |  |  |  |  |
|  | Current vs. Retired drivers | 0.499 | < 0.001 |  |  |  |  | 0.029 | 0.399 |  | 0.015 | 0.655 |  | 0.017 | 0.621 |  | 0.004 | 0.920 |  | 0.177 | < 0.001 |
|  | Current vs. Never drivers | 0.507 | < 0.001 |  |  |  |  | 0.075 | 0.007 |  | 0.093 | 0.001 |  | 0.081 | 0.003 |  | 0.063 | 0.026 |  | 0.182 | < 0.001 |
|  | Retired vs. Never drivers | 0.015 | 0.816 |  |  |  |  | 0.076 | 0.400 |  | 0.117 | 0.155 |  | 0.089 | 0.320 |  | 0.076 | 0.400 |  | 0.010 | 0.925 |
|  |  |  |  |  |  |  |  |  |  |  |  |  |  |  |  |  |  |  |  |  |  |
|  | Male vs. Female current drivers | 0.007 | 0.887 |  | 0.036 | 0.655 |  | 0.044 | 0.524 |  | 0.067 | 0.268 |  | 0.003 | 0.964 |  | 0.060 | 0.353 |  | 0.044 | 0.531 |
|  | Male vs. Female retired drivers | 0.008 | 0.903 |  |  |  |  | 0.190 | 0.465 |  | 0.284 | 0.212 |  | 0.012 | 0.964 |  | 0.187 | 0.471 |  | 0.118 | 0.754 |
|  | Male vs. Female never drivers | 0.021 | 0.853 |  |  |  |  | 0.129 | 0.643 |  | 0.150 | 0.534 |  | 0.075 | 0.823 |  | 0.062 | 0.835 |  | 0.014 | 0.964 |
|  |  |  |  |  |  |  |  |  |  |  |  |  |  |  |  |  |  |  |  |  |  |
| Volunteering | |  |  |  |  |  |  |  |  |  |  |  |  |  |  |  |  |  |  |  |  |
|  | Current vs. Retired drivers | 0.196 | < 0.001 |  |  |  |  | 0.078 | 0.060 |  | 0.084 | 0.038 |  | 0.130 | 0.001 |  | 0.026 | 0.602 |  | 0.086 | 0.034 |
|  | Current vs. Never drivers | 0.154 | < 0.001 |  |  |  |  | 0.146 | < 0.001 |  | 0.095 | 0.015 |  | 0.086 | 0.029 |  | 0.024 | 0.612 |  | 0.069 | 0.092 |
|  | Retired vs. Never drivers | 0.071 | 0.165 |  |  |  |  | 0.067 | 0.486 |  | 0.008 | 0.943 |  | 0.078 | 0.401 |  | 0.078 | 0.401 |  | 0.034 | 0.717 |
|  |  |  |  |  |  |  |  |  |  |  |  |  |  |  |  |  |  |  |  |  |  |
|  | Male vs. Female current drivers | 0.056 | 0.272 |  | 0.094 | 0.274 |  | 0.039 | 0.758 |  | 0.163 | 0.020 |  | 0.030 | 0.823 |  | 0.126 | 0.095 |  | 0.018 | 0.915 |
|  | Male vs. Female retired drivers | 0.026 | 0.853 |  |  |  |  | 0.125 | 0.758 |  | 0.135 | 0.754 |  | 0.016 | 0.964 |  | 0.234 | 0.437 |  | 0.223 | 0.450 |
|  | Male vs. Female never drivers | 0.055 | 0.602 |  |  |  |  | 0.099 | 0.758 |  | 0.132 | 0.643 |  | 0.036 | 0.943 |  | 0.208 | 0.356 |  | 0.038 | 0.942 |

Effect size (Cramer’s V) of <0.10 indicates negligible, <0.20 small, <0.50 medium, and ≧0.50 large differences.
