# Supplementary material for: Sex Differences in Instrumental Activities of Daily Living and Transportation Modes by Driving Status Among Older Adults
Source: Occup Ther Int. 2026 May 9;2026:6621910. doi: 10.1155/oti/6621910 (PMC13156801; doi:10.1155/oti/6621910)
Supplement: Supplementary file 3 — Supporting Information 3 Appendix C: Multivariable ordinal logistic regression analyses of IADL engagement frequency. [file OTI-2026-6621910-s003.docx]

| APPENDIX C. Multivariable ordinal logistic regression analyses of IADL engagement frequency. Ordinal logistic regression models (cumulative logit) were applied for each IADL outcome. The dependent variables were ordinal frequency categories ranging from “not at all” to “6–7 times per week.” All models were adjusted for driving status (current, retired, never), age, sex, household size, availability of family or acquaintances to provide rides, need for long-term care, and walkability index. Odds ratios (ORs) were calculated by exponentiating regression coefficients. P-values were derived from Wald tests, and Holm-adjusted p-values were calculated to account for multiple testing across models. Model fit was assessed using likelihood ratio (LR) tests, and Holm-adjusted p-values (LRT_Holm) were calculated to account for multiple testing across models. β, regression coefficient; SE, standard error; OR, odds ratio; CI, confidence interval; IADL, instrumental activities of daily living. | | | | | | |
| --- | --- | --- | --- | --- | --- | --- |
|  |  |  |  |  |  |  |
| Outcome | Model fit |  |  |  |  |  |
| Shopping for daily necessities | χ²(8) = 113.292, p < 0.001, Holm p < 0.001 |  |  |  |  |  |
|  | variable | β | SE | OR | OR_95%CI_low | OR_95%CI_high |
|  | Driving status: retired vs current (ref: current) | -0.754 | 0.148 | 0.471 | 0.352 | 0.629 |
|  | Driving status: never vs current (ref: current) | -0.618 | 0.153 | 0.539 | 0.4 | 0.727 |
|  | Age (per 1 year) | -0.106 | 0.029 | 0.899 | 0.849 | 0.952 |
|  | Sex: men vs women (ref: women) | -0.187 | 0.119 | 0.83 | 0.657 | 1.047 |
|  | Household size (per 1 person) | -0.011 | 0.057 | 0.989 | 0.885 | 1.105 |
|  | Availability of family or acquaintances to drive: yes vs no (ref: no) | -0.193 | 0.121 | 0.825 | 0.65 | 1.046 |
|  | Long-term care: yes vs no (ref: no) | -1.105 | 0.199 | 0.331 | 0.224 | 0.49 |
|  | Walkability Index (per 1 unit) | 0.463 | 0.123 | 1.589 | 1.247 | 2.024 |
|  |  |  |  |  |  |  |
| Visiting medical facilities | χ²(8) = 26.013, p = 0.001, Holm p = 0.001 |  |  |  |  |  |
|  | variable | β | SE | OR | OR_95%CI_low | OR_95%CI_high |
|  | Driving status: retired vs current (ref: current) | 0.067 | 0.151 | 1.069 | 0.795 | 1.437 |
|  | Driving status: never vs current (ref: current) | -0.104 | 0.163 | 0.902 | 0.655 | 1.24 |
|  | Age (per 1 year) | 0.032 | 0.03 | 1.033 | 0.973 | 1.096 |
|  | Sex: men vs women (ref: women) | -0.089 | 0.124 | 0.915 | 0.718 | 1.167 |
|  | Household size (per 1 person) | 0.016 | 0.058 | 1.017 | 0.907 | 1.14 |
|  | Availability of family or acquaintances to drive: yes vs no (ref: no) | -0.058 | 0.126 | 0.943 | 0.737 | 1.207 |
|  | Long-term care: yes vs no (ref: no) | 0.884 | 0.208 | 2.421 | 1.611 | 3.637 |
|  | Walkability Index (per 1 unit) | 0.225 | 0.127 | 1.252 | 0.976 | 1.607 |
|  |  |  |  |  |  |  |
| Leisure activities | χ²(8) = 36.514, p < 0.001, Holm p < 0.001 |  |  |  |  |  |
|  | variable | β | SE | OR | OR_95%CI_low | OR_95%CI_high |
|  | Driving status: retired vs current (ref: current) | 0.078 | 0.139 | 1.081 | 0.823 | 1.42 |
|  | Driving status: never vs current (ref: current) | 0.063 | 0.147 | 1.065 | 0.798 | 1.42 |
|  | Age (per 1 year) | -0.077 | 0.028 | 0.926 | 0.877 | 0.977 |
|  | Sex: men vs women (ref: women) | 0.335 | 0.113 | 1.398 | 1.119 | 1.745 |
|  | Household size (per 1 person) | -0.053 | 0.053 | 0.949 | 0.855 | 1.053 |
|  | Availability of family or acquaintances to drive: yes vs no (ref: no) | 0.311 | 0.119 | 1.365 | 1.082 | 1.723 |
|  | Long-term care: yes vs no (ref: no) | -0.403 | 0.189 | 0.668 | 0.462 | 0.968 |
|  | Walkability Index (per 1 unit) | 0.308 | 0.118 | 1.36 | 1.078 | 1.716 |
|  |  |  |  |  |  |  |
| Eating out | χ²(8) = 64.148, p < 0.001, Holm p < 0.001 |  |  |  |  |  |
|  | variable | β | SE | OR | OR_95%CI_low | OR_95%CI_high |
|  | Driving status: retired vs current (ref: current) | -0.547 | 0.146 | 0.578 | 0.434 | 0.77 |
|  | Driving status: never vs current (ref: current) | -0.586 | 0.154 | 0.556 | 0.412 | 0.752 |
|  | Age (per 1 year) | -0.05 | 0.029 | 0.951 | 0.899 | 1.006 |
|  | Sex: men vs women (ref: women) | -0.166 | 0.117 | 0.847 | 0.673 | 1.065 |
|  | Household size (per 1 person) | -0.127 | 0.055 | 0.881 | 0.79 | 0.982 |
|  | Availability of family or acquaintances to drive: yes vs no (ref: no) | 0.556 | 0.122 | 1.744 | 1.372 | 2.217 |
|  | Long-term care: yes vs no (ref: no) | -0.174 | 0.191 | 0.841 | 0.578 | 1.222 |
|  | Walkability Index (per 1 unit) | 0.383 | 0.123 | 1.466 | 1.153 | 1.865 |
|  |  |  |  |  |  |  |
| visiting someone's house | χ²(8) = 142.276, p < 0.001, Holm p < 0.001 |  |  |  |  |  |
|  | variable | β | SE | OR | OR_95%CI_low | OR_95%CI_high |
|  | Driving status: retired vs current (ref: current) | -1.191 | 0.15 | 0.304 | 0.227 | 0.408 |
|  | Driving status: never vs current (ref: current) | -0.773 | 0.156 | 0.461 | 0.34 | 0.626 |
|  | Age (per 1 year) | -0.062 | 0.029 | 0.94 | 0.888 | 0.994 |
|  | Sex: men vs women (ref: women) | -0.495 | 0.118 | 0.61 | 0.484 | 0.768 |
|  | Household size (per 1 person) | -0.083 | 0.056 | 0.92 | 0.825 | 1.026 |
|  | Availability of family or acquaintances to drive: yes vs no (ref: no) | 0.704 | 0.123 | 2.022 | 1.59 | 2.572 |
|  | Long-term care: yes vs no (ref: no) | -0.573 | 0.201 | 0.564 | 0.38 | 0.836 |
|  | Walkability Index (per 1 unit) | 0.198 | 0.127 | 1.218 | 0.951 | 1.561 |
|  |  |  |  |  |  |  |
| hobby | χ²(8) = 98.704, p < 0.001, Holm p < 0.001 |  |  |  |  |  |
|  | variable | β | SE | OR | OR_95%CI_low | OR_95%CI_high |
|  | Driving status: retired vs current (ref: current) | -0.887 | 0.147 | 0.412 | 0.309 | 0.549 |
|  | Driving status: never vs current (ref: current) | -0.996 | 0.155 | 0.369 | 0.272 | 0.501 |
|  | Age (per 1 year) | 0.027 | 0.028 | 1.027 | 0.972 | 1.086 |
|  | Sex: men vs women (ref: women) | -0.199 | 0.116 | 0.819 | 0.653 | 1.028 |
|  | Household size (per 1 person) | -0.102 | 0.054 | 0.903 | 0.812 | 1.004 |
|  | Availability of family or acquaintances to drive: yes vs no (ref: no) | 0.265 | 0.12 | 1.304 | 1.031 | 1.648 |
|  | Long-term care: yes vs no (ref: no) | -0.624 | 0.202 | 0.536 | 0.361 | 0.795 |
|  | Walkability Index (per 1 unit) | -0.08 | 0.116 | 0.923 | 0.736 | 1.158 |
